# Photocrosslinked Alginate-Methacrylate Hydrogels with Modulable Mechanical Properties: Effect of the Molecular Conformation and Electron Density of the Methacrylate Reactive Group

**DOI:** 10.3390/ma13030534

**Published:** 2020-01-22

**Authors:** Fernanda Araiza-Verduzco, Eustolia Rodríguez-Velázquez, Harold Cruz, Ignacio A. Rivero, Delvis R. Acosta-Martínez, Georgina Pina-Luis, Manuel Alatorre-Meda

**Affiliations:** 1 Tecnológico Nacional de México/I. T. Tijuana. Centro de Graduados e Investigación en Química-Grupo de Biomateriales y Nanomedicina, Blvd. Alberto Limón Padilla S/N, Tijuana 22510, BC, Mexico; fernandalynx@gmail.com (F.A.-V.); delvisrafael.acosta@gmail.com (D.R.A.-M.); 2 Facultad de Odontología, Universidad Autónoma de Baja California, Campus Tijuana, Calzada Universidad 14418, Tijuana 22390, BC, Mexico; 3 Tecnológico Nacional de México/I. T. Tijuana. Centro de Graduados e Investigación en Química, Blvd. Alberto Limón Padilla S/N, Tijuana 22510, BC, Mexico; harold.cruz@tectijuana.edu.mx (H.C.); irivero@tectijuana.mx (I.A.R.); gpinaluis@tectijuana.mx (G.P.-L.); 4 Cátedras CONACyT-Tecnológico Nacional de México/I. T. Tijuana. Centro de Graduados e Investigación en Química-Grupo de Biomateriales y Nanomedicina, Blvd. Alberto Limón Padilla S/N, Tijuana 22510, BC, Mexico

**Keywords:** alginate hydrogels, photocrosslinked hydrogels, methacrylated polysaccharides, mechanical properties, DFT calculations

## Abstract

Hydrogels for load-bearing biomedical applications, such as soft tissue replacement, are required to be tough and biocompatible. In this sense, alginate-methacrylate hydrogels (H-ALGMx) are well known to present modulable levels of elasticity depending on the methacrylation degree; however, little is known about the role of additional structural parameters. In this work, we present an experimental-computational approach aimed to evaluate the effect of the molecular conformation and electron density of distinct methacrylate groups on the mechanical properties of photocrosslinked H-ALGMx hydrogels. Three alginate-methacrylate precursor macromers (ALGMx) were synthesized: alginate-glycidyl methacrylate (ALGM1), alginate-2-aminoethyl methacrylate (ALGM2), and alginate-methacrylic anhydride (ALGM3). The macromers were studied by Fourier-transform infrared spectroscopy (FTIR), proton nuclear magnetic resonance (^1^H-NMR), and density functional theory method (DFT) calculations to assess their molecular/electronic configurations. In parallel, they were also employed to produce H-ALGMx hydrogels, which were characterized by compressive tests. The obtained results demonstrated that tougher hydrogels were produced from ALGMx macromers presenting the C=C reactive bond with an outward orientation relative to the polymer chain and showing free rotation, which favored in conjunction the covalent crosslinking. In addition, although playing a secondary role, it was also found that the presence of acid hydrogen atoms in the methacrylate unit enables the formation of supramolecular hydrogen bonds, thereby reinforcing the mechanical properties of the H-ALGMx hydrogels. By contrast, impaired mechanical properties resulted from macromer conditions in which the C=C bond adopted an inward orientation to the polymer chain accompanied by a torsional impediment.

## 1. Introduction

Hydrogels are a special class of biomaterials that has attracted great interest in research and clinical communities for their use in the biomedical field, in particular for advanced applications such as tissue engineering and regenerative medicine (TERM) [1]. Hydrogels are hydrophilic networks formed upon crosslinking of polymer chains, capable of absorbing water or biological fluids while maintaining their crosslinked structure [2]. The interest in these materials, both as 2D and 3D systems, stems from the fact that they closely resemble the natural environment of cells, allowing them to replicate and unravel diverse cell-extracellular matrix (cell-ECM) interactions [3,4,5].

Hydrogels for biomedical applications can be produced from natural polysaccharides [6], such as cellulose [7], chitosan (CH) [3,4], sodium alginate (ALG) [5,8], and dextran [9], and also from some synthetic polymers such as poly(acrylamide) (PAM) [10], poly(ethylene glycol) (PEG) [11,12], and poly(vinyl alcohol) (PVA) [13], among others. With respect to the synthesis methodologies, they can be produced by either physical or chemical crosslinking or even by the combination of both [14]. Examples of physical crosslinking processes include molecular entanglements of polymeric chains and/or interplay of secondary forces like ionic attraction and hydrogen bonding, as well as hydrophobic interactions [15]. Meanwhile, a representative strategy prompting the covalent crosslinking is photopolymerization [16,17,18]. Photopolymerization is a fast, mild, and reproducible approach based on the use of ultraviolet or visible light to promote the chemical reaction between photosensitive monomeric units in the presence of appropriate photoinitiators [4,5,19]. The use of photopolymerization in the preparation of hydrogels is advantageous in comparison with other crosslinking methods because liquid hydrogel precursors can be delivered and crosslinked in situ to form the hydrogels in a minimally invasive manner [17]. The process also allows the fabrication of hydrogels with complex shapes and modulable mechanical/physicochemical properties. Last but not least, photocrosslinked hydrogels can be designed to degrade via hydrolytic or enzymatic processes and, more importantly, to present biofunctional moieties within their structure to control cellular responses and/or initiate organ-specific tissue formation [18].

Of interest to this article, photocrosslinked ALG hydrogels have been prepared by grafting and subsequent photopolymerization of different methacrylate units [20,21], giving rise to systems that can withstand significant levels of stress without failure [17]. In this context, the role of the methacrylation degree on the mechanical behavior of these and similar materials has been thoroughly studied [17,22]. However, little or no attention has been paid to other factors governing the crosslinking process and the resulting mechanical properties, such as the molecular/electronic configuration of the grafted methacrylate group.

Filling this gap, we herein present an experimental-computational approach aimed to elucidate the possible correlation (if any) between the mechanical properties of photocrosslinked alginate-methacrylate hydrogels with the molecular conformation and electron density of different methacrylate reactive groups. To this end, three methacrylate groups with varying chain lengths and electron densities were grafted onto ALG, namely glycidyl methacrylate, 2-aminoethyl methacrylate, and methacrylic anhydride. The resulting alginate-methacrylate precursor macromers (ALGMx) were studied by FTIR, ^1^H-NMR, and DFT calculations and were also used to produce photocrosslinked alginate-methacrylate hydrogels (H-ALGMx). The obtained hydrogels were characterized by mechanical tests, and differences in the derived mechanical properties were explained by the theoretical calculations. Very importantly, the synthesis of each macromer was optimized beforehand to guarantee the required chemical structures and equivalent methacrylation degrees in all cases. Likewise, the experimental conditions of the photocrosslinking reactions were ensured to be the same in all cases. The obtained results are described below.

## 2. Materials and Methods

### 2.1. Materials

Sodium alginate (ALG, 120–180 kDa; Mannuronate/Guluronate ratio = 1.42), glycidyl methacrylate (M1, 97%), 2-aminoethyl methacrylate hydrochloride (M2, 90%) methacrylic anhydride (M3, ~99%), 2-hydroxy4′-(2-hydroxyethoxy)-2-methylpropiophenone (Irgacure 2959 or I2959, 98%), N-hydroxysuccinimide (NHS, 99%), 1-[3-(Dimethylamino)-propyl]-3-ethylcarbodiimide methiodide (EDC), sodium hydroxide (NaOH), acetic acid, and deuterium oxide (D2O) were purchased from Sigma-Aldrich and used as received. The guluronate proportion of ALG was assessed by ^1^H-NMR spectroscopy (see Section 3). Sterile filtered Milli-Q water (Direct-Q 3 UV) was used throughout.

### 2.2. Synthesis of Alginate-Glycidyl Methacrylate (ALGM1)

ALGM1 was synthesized as reported elsewhere [23], although with some optimizations. Briefly, ALG was dissolved into 100 mL of Milli-Q water to produce a 2% (w/v) solution. Then, 5.5 mL of M1 were added to the system and the solution was maintained under continuous stirring at 60 °C for 4 h, adjusting periodically the pH to 3 using concentrated acetic acid (every 20 min). The resulting solution was poured into 500 mL of cold ethanol to precipitate the ALGM1 product. Finally, the precipitate was vacuum filtered, washed three times with ethanol, air-dried, and stored at −20 °C until use. From the addition of M1, all the synthesis steps were carried out under dark conditions.

### 2.3. Synthesis of Alginate-2-Aminoethyl Methacrylate (ALGM2)

ALGM2 was synthesized as reported elsewhere [22]. Briefly, ALG was dissolved into 100 mL of MES buffer solution containing 0.5 M NaCl (pH 6.5) to produce a 1% (w/v) solution. Then, 0.53 g of NHS and 1.75 g of EDC were added to the mixture to activate the ALG carboxylic acid groups. After 5 min, 0.76 g of M2 were added to the system and the solution was maintained under continuous stirring at room temperature (RT) for 24 h. The resulting solution was poured into 500 mL of cold acetone to precipitate the ALGM2 product. Finally, the precipitate was vacuum filtered, washed three times with acetone, dialyzed for 3 d, freeze-dried, and stored at −20 °C until use. From the addition of M2, all the synthesis steps were carried out under dark conditions.

### 2.4. Synthesis of Alginate-Methacrylic Anhydride (ALGM3)

ALGM3 was synthesized as reported elsewhere [24]. Briefly, ALG was dissolved into 20 mL of Milli-Q water to produce a 2% (w/v) solution. Then, 20 mL of M3 were added to the system and the solution was maintained under continuous stirring for 3 d at R.T., adjusting periodically the pH to 7 using a NaOH aqueous solution (5 M). After the 3-day reaction period, the resulting solution was poured into 100 mL of cold ethanol to precipitate the ALGM3 product. Finally, the precipitate was vacuum filtered, washed three times with ethanol, air-dried, and stored at −20 °C until use. From the addition of M3, all the synthesis steps were carried out under dark conditions.

### 2.5. Synthesis of H-ALGMx Hydrogels

Each ALGMx macromer (ALGM1, ALGM2, and ALGM3) was dissolved separately in Milli-Q water to achieve a concentration of 2.5% (w/v) inside borosilicate vials. The resulting solutions were added with I2959 (10% (w/w) with respect to each macromer) and subjected to UV-light irradiation for 1 h (365 nm).

### 2.6. FTIR Characterization

FTIR spectra of all studied samples were recorded between 4000 and 650 cm^−1^ using a Perkin-Elmer/Spectrum 400 spectrophotometer fitted with a universal ATR sampling accessory.

### 2.7. ^1^H-NMR Characterization

^1^H-NMR spectra of all studied samples were acquired using a 9.4 T Bruker Avance III spectrometer operating at a 400 MHz proton frequency. The spectra were processed with the ACD labs processor software. All the samples were dissolved to 1% (w/v) in D_2_O. The G proportion in the ALG chain and the degree of methacrylation of the ALGMx macromers (also referred to as the degree of substitution or DS) were calculated from each corresponding spectrum according to Equations (1) and (2), respectively [25],

(1) $$G\;\left(\%\right)=\frac{H_{G-1}}{H_{M-1}+H_{G-1}}\times 100$$

(2) $$\mathrm{DS}\;\left(\%\right)=\frac{\frac{\mathrm{Ha}+\mathrm{Hb}}{2}}{H_{G-1}}\times G$$

where H_G-1_, H_M-1_, Ha, and Hb stand for the area under the curve of the anomeric carbon hydrogen in the guluronic units (ca. 4.80–5.20 ppm), the anomeric carbon hydrogen in the mannuronic units (ca. 4.50 ppm), and the two vinyl hydrogens in the methacrylate group (5.20–6.50 ppm), respectively [25].

### 2.8. Mechanical Characterization

Compressive tests were carried out employing a mechanical texturometer (Brookfield CT3-10Kg, AMETEK Brookfield, Middleboro, MA, USA) equipped with a cylindrical probe (TA11/1000, 25.4 mm D, 35 mm H). Briefly, freshly prepared hydrogels (6.75 mm in diameter) were mounted on top of a flat table and compressed up to 80% strain. The probe was programmed to descend at a fixed speed of 0.5 mm/s. The assay was carried out at RT (ca. 25 °C). The Young modulus (E), compressive strength (σ_F_), and toughness modulus (U_T_) were determined from the stress-strain curves. E was calculated as the slope of the curve along the elastic region (up to ε = 15%). σ_F_ corresponds to the value of the applied stress at the fracture point. Finally, U_T_ was calculated as the area under the whole curve (0 ≤ σ ≤ σ_F_).

### 2.9. Computational Calculations

The molecular structure, vibrational frequencies, and energies of the optimized geometries of the ALGMx macromers were computed by means of the density functional theory method (DFT), using the Gaussian 09 software package [26] with the B3LYP exchange-correlation functional [27] and the STO-3G* basis set [28]. 

### 2.10. Statistical Analysis

Statistical analysis was performed by the one-way analysis of variance (one-way ANOVA) for repeated measurements. The post hoc Tukey test was used to perform multiple comparisons and differences were considered significant at a level of *p* < 0.05.

## 3. Results and Discussion

### 3.1. Synthesis of Macromers and ^1^H-NMR Characterization

Glycidyl methacrylate (M1), 2-aminoethyl methacrylate (M2), and methacrylic anhydride (M3) were employed to functionalize ALG aiming to obtain ALGMx macromers with noticeable structural and electronic differences that could have an effect on the mechanical properties of H-ALGMx hydrogels thereupon produced. The selected methacrylate groups have been indistinctively used in the synthesis of ALG derivatives for biomedical applications [17,22,23,24,29,30].

**Figure 1:**
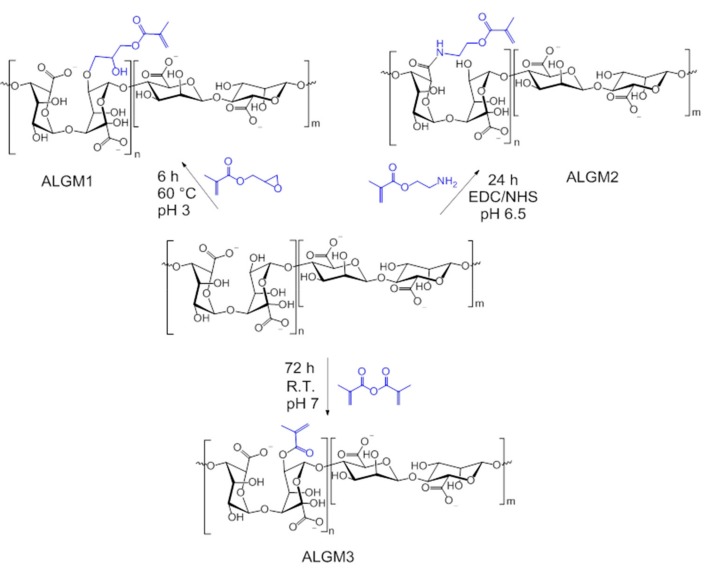
Reaction schemes of the alginate-methacrylate precursor macromers (ALGMx).

Figure 1 shows the reaction schemes of the synthesized macromers at optimized conditions. As portrayed in this figure and confirmed by the ^1^H-NMR spectra of each macromer (see below), the grafting of ALG with M1, M2, and M3 proceeded by well-known mechanisms of epoxide ring-opening, carbodiimide chemistry, and transesterification, respectively. As a result, ALGM1 and ALGM2 turned out to coincide in the same number of spacing atoms between the C=C reactive bond and the polymer chain (seven atoms), but having a different electron density resulting from the presence of four electronegative heteroatoms located at different positions of their respective Mx group. Meanwhile, ALGM3 proved to present a lower electron density (two heteroatoms) and a shorter number of spacing atoms (three atoms) with respect to their counterparts.

**Figure 2:**
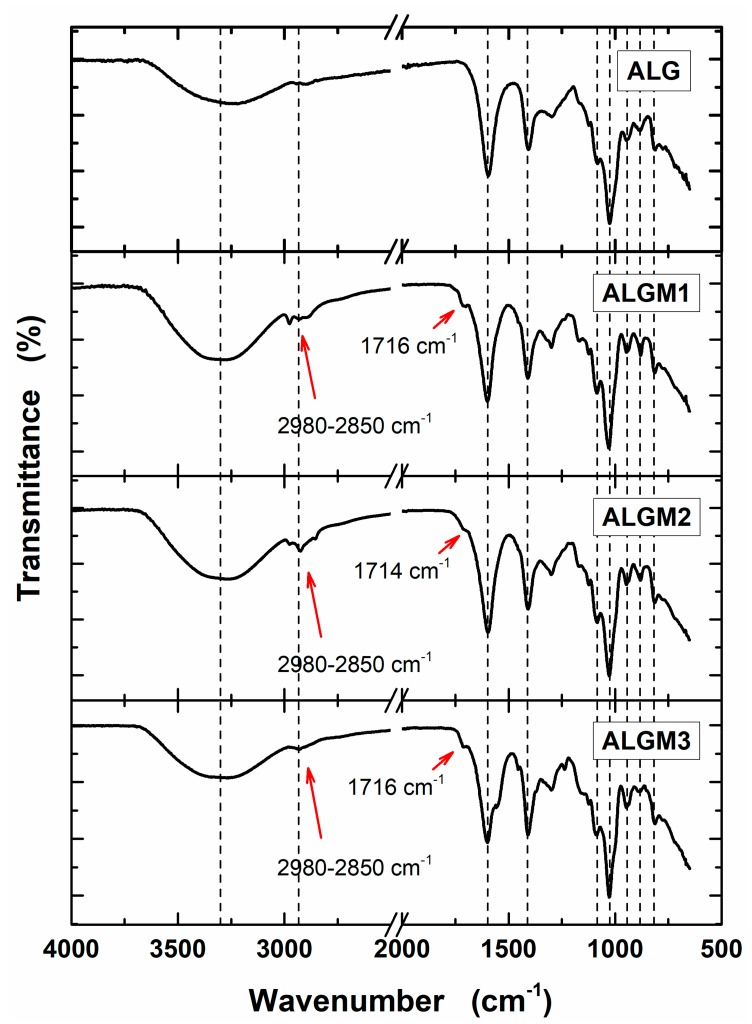
FTIR spectra of sodium alginate (ALG) and ALGMx macromers. The dotted lines mark bands common to all spectra. The red arrows mark the bands of the aliphatic chains (2980–2850 cm^−1^) and carboxyl esters (ca. 1715 cm^−1^) of the grafted Mx groups.

Native ALG and synthesized ALGMx macromers were characterized by FTIR and ^1^H-NMR to assess their chemical structure. Figure 2 shows the obtained FTIR spectra. As observed from this figure, all spectra share a set of nine bands (marked with dotted lines). The first two bands, located in the regions of 3700–3000 and 2980–2850 cm^−1^, are common to all polysaccharides and correspond to the stretching of –OH and –CH groups, respectively [31]. Meanwhile, the following seven bands, distinctive of ALG, are assigned to the stretching of carboxylate anions (COO– ca. 1600 cm^−1^) [31,32], to the C–OH deformation with contribution of O–C–O symmetric stretching of carboxylate groups (ca. 1406 cm^−1^) [32,33], to the C–O (ca. 1083 cm^−1^) and C–C (ca. 1025 cm^−1^) stretching vibrations of the pyranose rings [31], to the C–O stretching of uronic acid residues (ca. 943 cm^−1^) [31], to the C1–H deformation of β-mannuronic acid residues (ca. 879 cm^−1^) [31], and to C–H bending vibrations of mannuronic acid residues (ca. 817 cm^−1^) [34]. In addition to these bands, on the other hand, the spectra of the macromers exhibit the growth of the –CH stretching bands (2980–2850 cm^−1^) and the appearance of a shoulder around 1715 cm^−1^ (next to the COO– band), not observed in the ALG spectrum (marked with arrows). These growing bands and appearing shoulder can respectively be attributed to the stretching vibrations of the –CH groups of the aliphatic chains and the C=O group of the esters, both resulting from the grafting of the methacrylate units (see chemical structures in Figure 1). Accordingly, these bands provide evidence of the desired ALG functionalization with the selected Mx groups.

**Figure 3:**
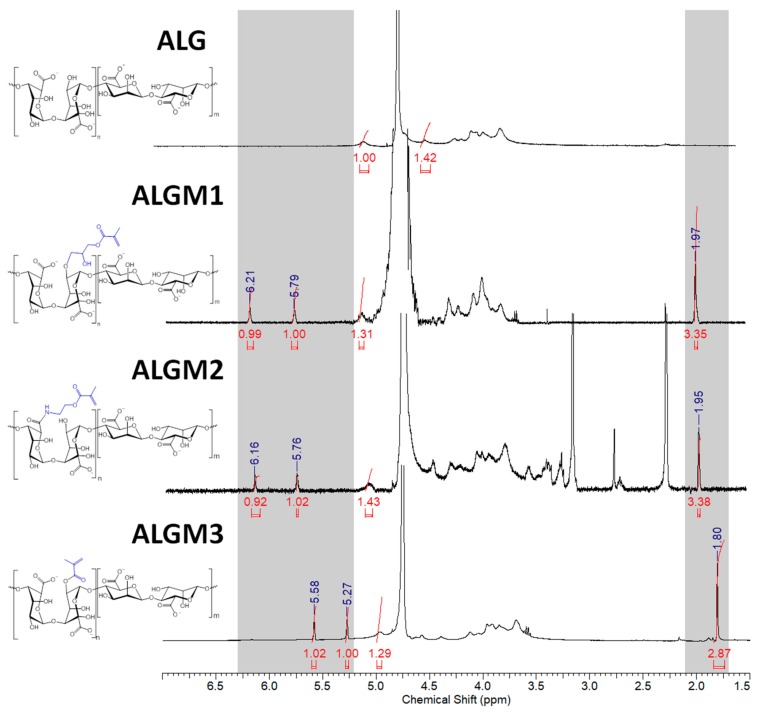
^1^H-NMR spectra of ALG and ALGMx macromers. The grey zones mark the chemical shift ranges of the vinyl (5.20–6.30 ppm) and methyl hydrogens (1.70–2.10 ppm) of the grafted Mx groups. D_2_0 was used as a solvent in all cases.

Figure 3 shows the ^1^H-NMR spectra of native ALG and the synthesized ALGMx macromers. As expected, all spectra display characteristic peaks between 3.50 and 5.20 ppm corresponding to the saccharide units of the ALG backbone. In addition, the macromers’ spectra exhibit the distinctive signals of the vinyl (5.20–6.30 ppm) and methyl hydrogens of the methacrylate grafted groups (ca. 2.00 ppm), which vary in location depending on the resulting chemical environment. In good agreement with previous publications, the signals corresponding to the vinyl hydrogens of the Mx groups appeared as two well-defined singlets at 5.79 and 6.21, 5.76 and 6.16, and 5.27 and 5.58 ppm for ALGM1 [23], ALGM2 [22], and ALGM3 [35], respectively. Meanwhile, the signal ascribed to the methyl hydrogens of each Mx appeared as a well-defined singlet at 1.97, 1.95, and 1.80 ppm for the macromers discussed in the very same order, respectively.

**Figure 4:**
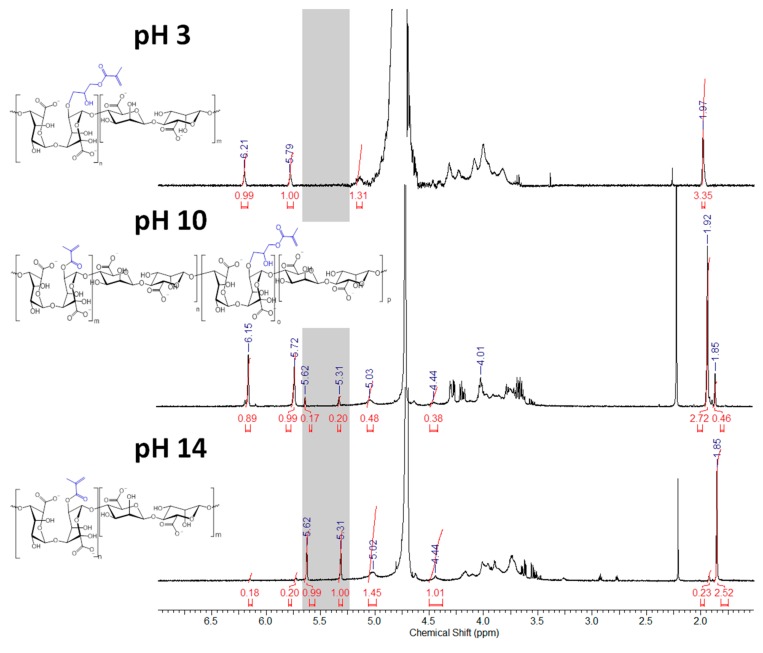
^1^H-NMR spectra of ALGMx macromers obtained by the grafting of M1 at varying reaction pHs. The gray zone marks the chemical shift range of the shielded vinyl hydrogens resulting from the grafting via the methacryloyl subunits (5.25–5.65 ppm). D20 was used as a solvent in all cases.

Interestingly, the vinyl signals of the ALGM1 and ALGM2 macromers showed up downfield with respect to those of ALGM3. This outcome can be attributed to the de-shielding effect of the oxygen and nitrogen atoms present in the alcohol and amide groups of M1 and M2. The oxygen and nitrogen atoms are not present in M3. Finally, the additional signals of the ALGM2 macromer correspond to the protons of the methylene group bound to the nitrogen of the amide (3.40 ppm) [22,36] and to the protons of the methyl (2.75–3.35 ppm) and methylene groups (2.30 ppm) of non-removed traces of (N-ethyl-N’-(3-dimethylaminopropyl) urea (EDU) [37], an EDC-urea residue commonly found in carbodiimide-driven reactions of ALG and other polysaccharides [22,38]. The protons of the methylene group bound to the oxygen of the ester are not distinguished in the spectrum since they overlap with the signals of the saccharide unit (at ca. 4.30 ppm) [36]. Worth mentioning, EDU is well known as a nonreversible, inert byproduct of the EDC activation [38,39]; therefore, it does not take part in any subsequent reaction of the given obtained product (e.g., the desired photocrosslinking reaction of ALGM2 in our case). Hence, the non-removed traces of EDU are not expected to play any role on the mechanical properties of the synthesized H-ALGM2 hydrogel (characterized below). On the other hand, with respect to the synthesis of ALGM1, it was found that a tight control of reaction pH to acidic conditions (pH 3) was mandatory given that the grafting of M1 onto ALG turned out to be regioselective. As shown in Figure 4, the rising of pH to basic conditions (pH 10 and 14) led to the formation of products exhibiting two well-defined singlets at 5.31 and 5.62 ppm, not observed for ALGM1 synthesized at pH 3. Considering that these signals lie in the region of the shielded vinyl hydrogens of ALGM3 and also that transesterification of M1 can occur as a parallel mechanism to the epoxide ring-opening already cited [40], it is very likely that the new signals correspond to the simultaneous (pH 10) or favored grafting (pH 14) of the M1 groups by their methacryloyl subunits, giving rise to either ALGM1-co-ALGM3 or ALGM3 macromers, respectively. Both of these last products were not of interest to this study, thus they were not further considered.

The G proportion in the ALG chain and the DS of the ALGMx macromers were calculated from the spectra shown in Figure 3 according to Equations (1) and (2), respectively (see Section 2) [25]. G was calculated as 41.32%, while the DS of each macromer was determined to amount to 31.39%, 28.03%, and 32.35% for ALGM1, ALGM2, and ALGM3, respectively.

### 3.2. Mechanical Characterization of H-ALGMx Hydrogels

The synthesized macromers were photocrosslinked in solution and the resulting H-ALGMx hydrogels were characterized by mechanical compressive tests. 

**Figure 5:**
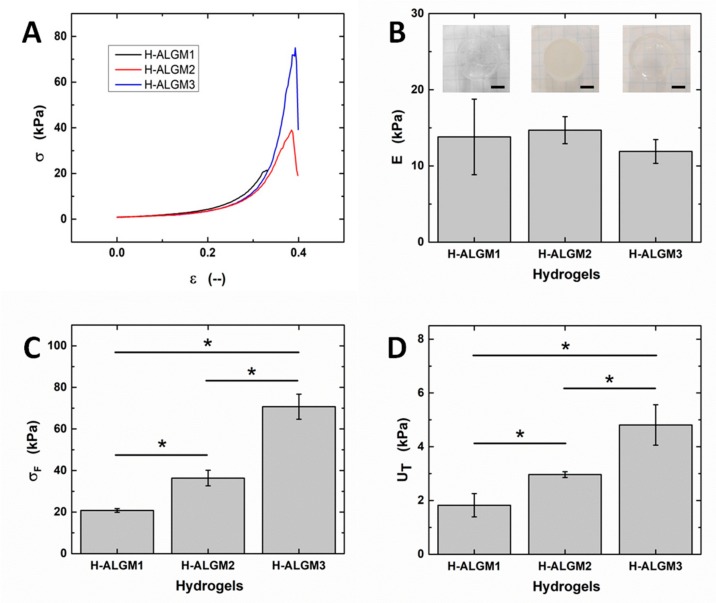
Mechanical properties of H-ALGMx hydrogels: (**A**) Compressive stress-strain curves, (**B**) Young moduli, (**C**) compressive strength, and (**D**) toughness moduli. Insets in (**B**) show representative photographs of each hydrogel, wherein the scale bars stand for 1 cm. As observed from the insets, H-ALGM1 and H-ALGM3 turned out to be transparent, while H-ALGM2 was found to be opaque. (Note the squared pattern beneath each hydrogel to appreciate its transparency or opacity).

Figure 5 shows the obtained stress-strain curves and the mechanical properties thereby derived. It can be observed from the curves (panel A) that all samples displayed the typical evolution of brittle materials subjected to stress, involving the elastic (ε ≤ 0.15), plastic (0.15 < ε ≤ ε_F_), and fracture deformations (ε > ε_F_), as described for other polysaccharide-based hydrogels [3]. In good agreement with other reports on hydrogels of similar nature [17,22], the Young’s moduli of the H-ALGMx hydrogels were determined to amount to ca. 15 kPa (panel B), although with no statistical difference among the tested samples, revealing that they undergo an equivalent elastic deformation upon application of the external stress. By contrast, the compressive strength and toughness moduli (panels C and D, respectively) were found to follow the same increasing trend as H-ALGM1 < H-ALGM2 < H-ALGM3. These apparently contradictory results can be understood if the fracture mechanism of crosslinked materials is analyzed. As explained in previous publications [3,41], during the elastic deformation, which is reversible, non-crosslinked chains (non-spatially restricted chains) align with the direction of the stress, compensating its effect to avoid a permanent deformation of the bulk material. If the imposed stress is maintained, then not only non-crosslinked, but hitherto motionless crosslinked chains also start to undergo spatial restructuring, up to a certain point in which a plastic deformation is reached (permanent deformation). At this stage of plastic deformation, the crosslinking density gains importance since the higher it is the tighter the junction between polymeric chains, thus the tougher the bulk material. Finally, if the plastic deformation is surpassed and the imposed stress is kept, then the stage of failure is observed, entailing the appearance of a fracture deformation and its propagation. Based on the aforementioned, and bearing in mind that the studied hydrogels were prepared at the very same experimental conditions, their similar response at the elastic region can be ascribed to the equivalent DS of the ALGMx macromers (ca. 30%, see the previous section), which resulted in a high and equivalent proportion of non-crosslinked chains or chain segments among the H-ALGMx hydrogels (those pertaining to the percentage of non-functionalized ALG, ca. 70% in all macromers). As above cited, the alignment of non-crosslinked chains with the direction of the imposed stress is the parameter preserving the reversible deformation of the bulk materials at this early stage, whereas the statistically different response of the hydrogels at the plastic region appears to be related to the chemical nature of each macromer, which has a clear effect on the mechanical toughness and maximum stress the systems can withstand without failing. As also outlined above, the density of crosslinked chains is the parameter governing the mechanical response of the tested materials at this stage, wherein the higher the crosslinking density the higher σ_F_ and U_T_ (see panels C and D). This hypothesis was validated by density functional calculations, as described below.

### 3.3. Density Functional Calculations

Computational calculations based on the DFT method were carried out to elucidate the spatial configuration and electron density of the synthesized macromers. DFT-based calculations have been highlighted as a valuable tool in organic chemistry to predict and/or explain the reactivity and derived physical properties of functional materials [42].

**Figure 6:**
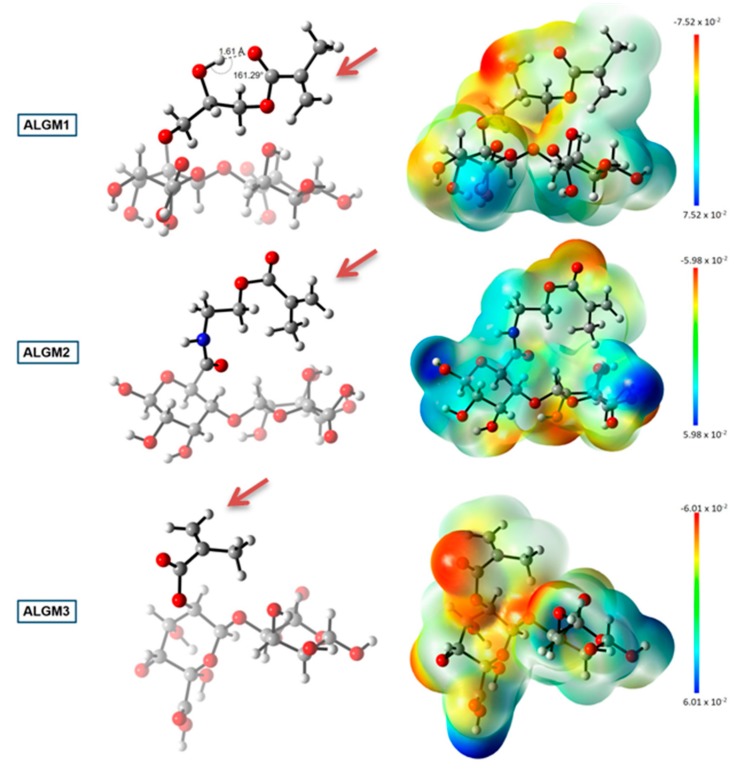
DFT calculations: geometry optimized structures (left) and electrostatic potential maps (right) of the ALGMx macromers. Carbon, hydrogen, oxygen, and nitrogen atoms are represented as gray, white, red, and blue spheres, respectively. Two sugar rings representing the ALG chain were considered in the calculations. The brown arrows mark the C=C reactive bonds. The bar at the far right of the image represents the color code of the isodensity surface. DFT coordinates and electronic energies are presented as Supplementary Materials.

Figure 6 shows the geometry optimized structures and the electrostatic potential maps of the ALGMx macromers, wherein two sugar rings were considered to represent the ALG chain. This figure reveals distinctive features of the methacrylate group of each macromer. First and foremost, it was observed that M3 adopts the most favorable conformation upon grafting to ALG (see the ALGM3 panel), showing an outward orientation with respect to the polymer chain and the absence of any observable intramolecular interaction that could impair its free rotation, thus leaving the C=C reactive bond totally available for the desired crosslinking reaction. By contrast, the methacrylate units of ALGM1 and ALGM2 have the C=C bond oriented in rather less favorable directions, that of M1 in an inward position and that of M2 in a parallel position to the polymer chain. These positions result from the folding of the methacrylate chains due to the presence of the electronegative heteroatoms. Obviously, the orientation of the C=C reactive bond in both cases, in particular for ALGM1, is anticipated to hamper its availability for the desired crosslinking reaction, as compared to ALGM3. Second, at the same time of showing free rotation (as observed for M3), it was identified that M2 presents an acid hydrogen (depicted in a light blue area) resulting from the removal of electron density by the inductive effect of the amide nitrogen. This acid hydrogen enables ALGM2 to form supramolecular hydrogen bonds with neighbor polymer chains [43], reinforcing to some extent the crosslinking joints expected to occur by the C=C reactive bond. And third, it was also found that ALGM1 presents an intramolecular hydrogen bond in its methacrylate unit (OH–O), displaying an in-between distance of 1.61 Å and an angle of 161.29°, which correspond to a rather strong hydrogen bonding [44]. This intramolecular interaction makes the grafted M1 group rigid, imposing an additional impediment to ALGM1 chains to react with one another [45].

Taking the above-mentioned information into consideration, the computational results might provide an explanation for the observed trend in the mechanical properties: H-ALGM1 < H-ALGM2 < H-ALGM3 (*p* < 0.05). On the one hand, they demonstrated the C=C bonds of the ALGM3 macromer as the ones most favorably oriented to establish a crosslinking reaction with their homologues, showing the additional advantage of being free to rotate. As a result, the H-ALGM3 hydrogel is the one expected to have the highest number of crosslinking joints (i.e., the highest crosslinking density), giving support to the uppermost mechanical properties it displayed. Next to ALGM3, ALGM2 has the second most available C=C bonds and also holds the possibility to set up reinforcing hydrogen bonds with neighbor polymer chains, explaining the formation of a softer H-ALGM2 hydrogel as compared to H-ALGM3, although a bit tougher as compared to H-ALGM1. Finally, H-ALGM1 proved to be the weakest hydrogel because of the poor orientation of their C=C reactive bonds coupled with a certain rigidity of the M1 unit, which hamper in ensemble the possibility of photocrosslinking to a high extent.

## 4. Conclusions

The study presented here portrays an experimental-computational characterization by which differences in compressive strength and toughness of photocrosslinked ALG-methacrylate hydrogels were assessed and explained. The obtained results suggest that changes in these mechanical properties can be ascribed to variations in the molecular conformation and electron density of the methacrylate reactive groups. In particular, it was observed that tougher hydrogels were those produced from ALGMx macromers having C=C bonds favorably oriented with respect to the polymer chain and free rotation. To the best of our knowledge, this is the first time a comprehensive study like this has been carried out to elucidate the effect of the methacrylate functional units on the mechanical performance of the resulting hydrogels. Thus, the presented approach might in our opinion be instructive to tackle the rational design of photocrosslinked hydrogels with modulable mechanical properties.

## Supplementary Materials

The following are available online at https://www.mdpi.com/1996-1944/13/3/534/s1, DFT coordinates and electronic energies.
