# Supplementary material for: Photocrosslinked Alginate-Methacrylate Hydrogels with Modulable Mechanical Properties: Effect of the Molecular Conformation and Electron Density of the Methacrylate Reactive Group
Source: Materials (Basel). 2020 Jan 22;13(3):534. doi: 10.3390/ma13030534 (PMC7040623; doi:10.3390/ma13030534)
Supplement: Supplementary file 1 [file materials-13-00534-s001.pdf]

materials

*Supplementary*

# Photocrosslinked Alginate–Methacrylate Hydrogels with Modulable Mechanical Properties: Effect of the Molecular Conformation and Electron Density of the Methacrylate Reactive Group

**Fernanda Araiza-Verduzco [1], Eustolia Rodríguez-Velázquez [1,2,*], Harold Cruz [3], Ignacio A. Rivero [3], Delvis R. Acosta-Martínez [1], Georgina Pina-Luis [3] and Manuel Alatorre-Meda [4,*]**

1 Tecnológico Nacional de México/I. T. Tijuana. Centro de Graduados e Investigación en Química-Grupo de Biomateriales y Nanomedicina, Blvd. Alberto Limón Padilla S/N, 22510 Tijuana, BC, México; fernandalynx@gmail.com (F.A.-V.); delvisrafael.acosta@gmail.com (D.R.A.-M.)
2 Facultad de Odontología, Universidad Autónoma de Baja California, Campus Tijuana, Calzada Universidad 14418, 22390 Tijuana, BC, México
3 Tecnológico Nacional de México/I. T. Tijuana. Centro de Graduados e Investigación en Química, Blvd. Alberto Limón Padilla S/N, 22510 Tijuana, BC, México; harold.cruz@tectijuana.edu.mx (H.C.); irivero@tectijuana.mx (I.A.R.); gpinaluis@tectijuana.mx (G.P.-L.)
4 Cátedras CONACyT-Tecnológico Nacional de México/I. T. Tijuana. Centro de Graduados e Investigación en Química-Grupo de Biomateriales y Nanomedicina, Blvd. Alberto Limón Padilla S/N, 22510 Tijuana, BC, México
* Correspondence: eustolia.rodriguez@uabc.edu.mx (E.R.-V.); malatorreme@conacyt.mx (M.A.-M.)

**XYZ coordinates and electronic energies of the calculated structures**

*ALGM1*

Zero−point correction = 0.206040 (Hartree/Particle)
Thermal correction to Energy = 0.219552
Thermal correction to Enthalpy = 0.220496
Thermal correction to Gibbs Free Energy = 0.164461
Sum of electronic and zero−point Energies = −604.751446
Sum of electronic and thermal Energies = −604.737934
Sum of electronic and thermal Enthalpies = −604.736990
Sum of electronic and thermal Free Energies = −604.793025

# opt freq b3lyp/sto−3g* geom = connectivity
XYZ coordinates

| | | | |
|---|---|---|---|
| O | 2.14664 | 4.38268 | −0.02585 |
| C | 1.33143 | 3.30916 | 0.34672 |
| C | −0.0722 | 3.36141 | −0.36027 |
| C | 1.97967 | 1.92387 | 0.08449 |
| H | 1.19844 | 3.47317 | 1.44273 |
| C | 1.02302 | 0.81197 | 0.6001 |
| H | 2.97153 | 1.84363 | 0.59091 |
| C | −0.3768 | 0.98167 | −0.06663 |
| H | 1.43221 | −0.1992 | 0.31184 |
| H | −0.34948 | 0.6027 | −1.12306 |
| O | 2.31242 | 1.72042 | −1.26764 |
| H | 1.57388 | 2.03329 | −1.7938 |
| O | 0.1545 | 3.23966 | −1.74437 |
| H | −0.65258 | 3.39467 | −2.2188 |

| | | | |
|---|---|---|---|
| O | −0.93082 | 2.28346 | −0.03571 |
| O | −1.4009 | 0.39091 | 0.69514 |
| C | −0.85835 | 4.62802 | 0.06168 |
| O | −0.94247 | 5.12485 | 1.16379 |
| O | −1.54279 | 5.24147 | −0.93282 |
| H | −1.99192 | 6.00958 | −0.59338 |
| O | 1.01449 | 0.91622 | 2.00285 |
| C | −2.16552 | −0.5895 | −0.0016 |
| C | −2.04471 | −1.95072 | 0.74536 |
| C | −3.65481 | −0.14546 | −0.04881 |
| H | −1.79552 | −0.69078 | −1.05707 |
| H | −2.38264 | −1.83613 | 1.80664 |
| C | −4.449 | −1.28008 | −0.75882 |
| H | −3.74457 | 0.80484 | −0.62888 |
| C | −4.21689 | −2.61681 | 0.0121 |
| H | −4.09378 | −1.36898 | −1.81535 |
| H | −4.70344 | −2.60515 | 1.01937 |
| O | −4.10229 | 0.06417 | 1.27104 |
| H | −4.94848 | 0.48537 | 1.20195 |
| O | −5.80933 | −0.91159 | −0.75487 |
| H | −6.27707 | −1.60715 | −1.19802 |
| O | −4.61593 | −3.67391 | −0.80089 |
| H | −4.64682 | −4.46349 | −0.27581 |
| O | −2.84115 | −2.94405 | 0.10716 |
| C | −0.59514 | −2.46528 | 0.72678 |
| O | 0.11043 | −2.42728 | 1.7234 |
| H | 2.43106 | 4.2451 | −0.92222 |
| H | 0.44467 | 0.23127 | 2.33034 |
| N | −0.03889 | −2.87056 | −0.51815 |
| H | −0.69766 | −3.28215 | −1.14258 |
| C | 1.29062 | −3.5284 | −0.55142 |
| H | 1.31225 | −4.23739 | −1.40341 |
| H | 1.47118 | −4.13291 | 0.36315 |
| C | 2.38529 | −2.46281 | −0.71276 |
| H | 2.29098 | −1.91515 | −1.66881 |
| H | 2.34024 | −1.70555 | 0.11179 |
| C | 4.76427 | −2.53413 | −0.33589 |
| O | 5.68872 | −3.3092 | −0.45249 |
| O | 3.62112 | −3.16581 | −0.7366 |
| C | 4.81257 | −1.12715 | 0.15021 |
| C | 5.32636 | −0.16231 | −0.61092 |
| H | 5.38045 | 0.87186 | −0.27845 |
| H | 5.71451 | −0.33645 | −1.61053 |
| C | 4.29477 | −0.89637 | 1.52347 |
| H | 4.37344 | 0.15802 | 1.82175 |
| H | 3.2288 | −1.18006 | 1.57712 |
| H | 4.84386 | −1.49759 | 2.25957 |

*ALGM2*

Zero−point correction = 0.219595 (Hartree/Particle)
Thermal correction to Energy = 0.233901
Thermal correction to Enthalpy = 0.234845

Thermal correction to Gibbs Free Energy = 0.175053
Sum of electronic and zero−point Energies = −585.210485
Sum of electronic and thermal Energies = −585.196179
Sum of electronic and thermal Enthalpies = −585.195235
Sum of electronic and thermal Free Energies = −585.255027

# opt freq b3lyp/sto−3g* geom = connectivity
XYZ coordinates

| | | | |
|---|---|---|---|
| O | 4.75918 | −1.3489 | 0.09802 |
| C | 3.55716 | −0.76194 | −0.46245 |
| C | 2.26972 | −1.72436 | −0.19716 |
| C | 3.27342 | 0.53673 | 0.40475 |
| H | 3.64006 | −0.56773 | −1.55299 |
| C | 1.82926 | 1.14279 | 0.12869 |
| H | 4.00844 | 1.33198 | 0.15301 |
| C | 0.70202 | 0.02712 | 0.17425 |
| H | 1.59853 | 1.91643 | 0.89268 |
| H | 0.53534 | −0.32062 | 1.23342 |
| O | 3.57274 | 0.07357 | 1.77073 |
| H | 2.93072 | −0.77871 | 1.80903 |
| O | 2.18316 | −1.95159 | 1.28211 |
| H | 2.65317 | −2.87088 | 1.33813 |
| O | 1.01547 | −1.15641 | −0.68515 |
| O | −0.52931 | 0.60866 | −0.36499 |
| C | 2.48117 | −3.14052 | −0.88501 |
| O | 2.28739 | −3.5203 | −2.0547 |
| O | 3.01672 | −3.97383 | 0.15133 |
| H | 3.14016 | −4.8965 | −0.27178 |
| O | 1.89598 | 1.72256 | −1.23433 |
| C | 1.61885 | 3.13674 | −1.32629 |
| O | 1.8934 | 3.69526 | −2.41159 |
| C | 0.93419 | 3.84566 | −0.11935 |
| C | 1.6187 | 4.71061 | 0.64502 |
| H | 2.68727 | 4.90521 | 0.49201 |
| H | 1.13547 | 5.26205 | 1.46134 |
| C | −0.56717 | 3.5508 | 0.03708 |
| H | −1.13096 | 4.04075 | −0.77508 |
| H | −0.74039 | 2.45668 | −0.04316 |
| H | −0.93986 | 3.92929 | 1.00065 |
| C | −1.65416 | −0.32936 | −0.11515 |
| C | −2.57447 | 0.16364 | 1.08529 |
| C | −2.51111 | −0.41307 | −1.43881 |
| H | −1.27876 | −1.34872 | 0.11797 |
| H | −2.9773 | 1.17176 | 0.8259 |
| C | −3.82919 | −1.25348 | −1.18959 |
| H | −1.89133 | −0.93553 | −2.20967 |
| C | −4.54814 | −0.72669 | 0.12091 |
| H | −3.63152 | −2.34569 | −1.09501 |
| H | −4.83769 | 0.35 | −0.05173 |
| O | −2.93966 | 0.91326 | −1.88475 |
| H | −3.79117 | 0.60234 | −2.39885 |
| O | −4.64968 | −0.92976 | −2.37272 |

| | | | |
|---|---|---|---|
| H | −5.60046 | −1.11279 | −2.03082 |
| O | −5.70108 | −1.58606 | 0.31699 |
| H | −6.16155 | −1.15412 | 1.12641 |
| O | −3.67587 | −0.80374 | 1.32011 |
| C | −1.83125 | 0.29799 | 2.47172 |
| O | −2.08003 | 1.13022 | 3.37174 |
| O | −0.81826 | −0.69995 | 2.61745 |
| H | −0.46782 | −0.51046 | 3.5661 |
| H | 4.66472 | −0.88475 | 1.04702 |

*ALGM3*

Zero−point correction = 0.131100 (Hartree/Particle)
Thermal correction to Energy = 0.139422
Thermal correction to Enthalpy = 0.140366
Thermal correction to Gibbs Free Energy = 0.097861
Sum of electronic and zero−point Energies = −341.182559
Sum of electronic and thermal Energies = −341.174237
Sum of electronic and thermal Enthalpies = −341.173293
Sum of electronic and thermal Free Energies = −341.215799

# opt freq b3lyp/sto−3g* guess = mix geom = connectivity
XYZ coordinates

| | | | |
|---|---|---|---|
| O | −5.77555 | −0.97724 | 0.32287 |
| C | −4.41191 | −0.92871 | 0.01255 |
| C | −3.937 | 0.54257 | −0.27706 |
| C | −3.524 | −1.64854 | 1.06584 |
| H | −4.36321 | −1.50622 | −0.94294 |
| C | −2.01898 | −1.4826 | 0.70113 |
| H | −3.7931 | −2.73136 | 1.093 |
| C | −1.7204 | 0.03476 | 0.51331 |
| H | −1.3783 | −1.85725 | 1.53974 |
| H | −1.78118 | 0.5544 | 1.50516 |
| O | −3.78657 | −1.27491 | 2.39349 |
| H | −3.64813 | −0.33856 | 2.47722 |
| O | −4.26539 | 1.34143 | 0.82827 |
| H | −4.13313 | 2.25565 | 0.61172 |
| O | −2.53687 | 0.69562 | −0.43244 |
| O | −0.50086 | 0.23989 | −0.15274 |
| C | −4.5296 | 1.04195 | −1.6238 |
| O | −4.7322 | 0.41159 | −2.63915 |
| O | −4.84457 | 2.35843 | −1.66388 |
| H | −5.20027 | 2.58145 | −2.51867 |
| O | −1.81448 | −2.24007 | −0.48544 |
| C | −0.67409 | −3.07344 | −0.49972 |
| C | 0.28295 | 1.33864 | 0.31598 |
| C | 1.50272 | 0.80221 | 1.12801 |
| C | 0.76927 | 2.13148 | −0.93033 |
| H | −0.31759 | 2.03418 | 0.95597 |
| H | 2.0733 | 0.01757 | 0.53022 |
| C | 1.72225 | 3.2555 | −0.43311 |
| H | −0.11194 | 2.57995 | −1.45001 |
| C | 2.87556 | 2.60454 | 0.38995 |

| | | | |
|---|---|---|---|
| H | 1.14365 | 3.97242 | 0.20065 |
| H | 3.55121 | 1.98159 | −0.25547 |
| O | 1.42858 | 1.23909 | −1.7995 |
| H | 1.47975 | 1.67311 | −2.6401 |
| O | 2.21333 | 3.92741 | −1.57096 |
| H | 2.77624 | 4.61947 | −1.24988 |
| O | 3.53834 | 3.60674 | 1.09371 |
| H | 4.26766 | 3.21722 | 1.55953 |
| O | 2.37983 | 1.86258 | 1.49025 |
| C | 1.03859 | 0.16385 | 2.43861 |
| O | 0.96251 | −1.03031 | 2.68532 |
| O | 0.62846 | 0.98791 | 3.42158 |
| H | 0.40604 | 0.48221 | 4.19818 |
| H | −5.89478 | −0.62254 | 1.19641 |
| H | −0.57692 | −3.63518 | 0.45043 |
| H | −0.96062 | −3.79021 | −1.29147 |
| C | 0.6244 | −2.30538 | −0.85642 |
| H | 0.40377 | −1.1996 | −0.95139 |
| C | 1.68334 | −2.54581 | 0.24122 |
| H | 1.30708 | −2.20188 | 1.24092 |
| H | 1.97716 | −3.60971 | 0.31504 |
| O | 1.07658 | −2.63039 | −2.14473 |
| O | 2.80243 | −1.6806 | 0.08197 |
| C | 3.88891 | −2.0923 | −0.64221 |
| O | 3.93436 | −3.21884 | −1.09224 |
| C | 4.94119 | −1.03794 | −0.74355 |
| C | 4.74575 | 0.05642 | −1.47727 |
| H | 5.50749 | 0.82371 | −1.59062 |
| H | 3.82115 | 0.25248 | −2.01704 |
| C | 6.19961 | −1.32084 | −0.00851 |
| H | 6.00891 | −1.42109 | 1.06854 |
| H | 6.65118 | −2.26021 | −0.35621 |
| H | 6.94216 | −0.52339 | −0.14328 |
| H | 1.99684 | −2.37252 | −2.2354 |

© 2020 by the authors. Submitted for possible open access publication under the terms and conditions of the Creative Commons Attribution (CC BY) license (http://creativecommons.org/licenses/by/4.0/).
